# Supplementary material for: Resolving the full spectrum of human genome variation using Linked-Reads
Source: Genome Res. 2019 Apr;29(4):635–45. doi: 10.1101/gr.234443.118 (PMC6442396; doi:10.1101/gr.234443.118)
Supplement: Supplemental Material [file supp_gr.234443.118_Supplemental_longranger-2.2.zip › longranger-2.2.tar.bz2!/lr2.2-public/lib/stan/cmdstan/stan/doc/api/html/operator__minus__equal_8hpp_source.html]

Stan: src/stan/agrad/rev/operators/operator\_minus\_equal.hpp Source File


|  |  |
| --- | --- |
|  | Stan  2.5.0  probability, sampling & optimization |


- Main Page
- Related Pages
- Namespaces
- Classes
- Files

- File List
- File Members

All Classes Namespaces Files Functions Variables Typedefs Enumerations Enumerator Friends Macros Pages

- src
- stan
- agrad
- rev
- operators

operator\_minus\_equal.hpp

Go to the documentation of this file.

1 #ifndef STAN\_\_AGRAD\_\_REV\_\_OPERATORS\_\_OPERATOR\_MINUS\_EQUAL\_HPP

2 #define STAN\_\_AGRAD\_\_REV\_\_OPERATORS\_\_OPERATOR\_MINUS\_EQUAL\_HPP

3

4 #include <stan/agrad/rev/var.hpp>

5 #include <stan/agrad/rev/operators/operator\_subtraction.hpp>

6

7 namespace stan {

8  namespace agrad {

9

10  inline var& var::operator-=(const var& b) {

11  vi\_ = new subtract\_vv\_vari(vi\_,b.vi\_);

12  return \*this;

13  }

14

15  inline var& var::operator-=(const double b) {

16  if (b == 0.0)

17  return \*this;

18  vi\_ = new subtract\_vd\_vari(vi\_,b);

19  return \*this;

20  }

21

22  }

23 }

24 #endif

stan::agrad::var::operator-=

var & operator-=(const var &b)

The compound subtract/assignment operator for variables (C++).

**Definition:** operator\_minus\_equal.hpp:10

operator\_subtraction.hpp

stan::agrad::var::vi\_

vari \* vi\_

Pointer to the implementation of this variable.

**Definition:** var.hpp:40

var.hpp

stan::agrad::var

Independent (input) and dependent (output) variables for gradients.

**Definition:** var.hpp:27


---

[ Stan Home Page ]

*© 2011–2014,
Stan Development Team.*
